# Effect of alginate concentration on chondrogenesis of co-cultured human adipose-derived stem cells and nasal chondrocytes: a biological study

**DOI:** 10.1186/s40824-017-0105-7

**Published:** 2017-10-17

**Authors:** Y. W. Ewa-Choy, B. Pingguan-Murphy, N. A. Abdul-Ghani, J. Jahendran, K. H. Chua

**Affiliations:** 1 Department of Physiology, Faculty of Medicine, Universiti Kebangsaan Malaysia, Kuala Lumpur, Malaysia; 2 Department of Biomedical Engineering, Faculty of Engineering, University of Malaya, Kuala Lumpur, Malaysia; 3 Department of Obstetrics and Gynaecology, Faculty of Medicine, Universiti Kebangsaan Malaysia, Kuala Lumpur, Malaysia; 4 Suite 16, Pantai Cheras Medical Centre, Kuala Lumpur, Malaysia

**Keywords:** Alginate concentration, Chondrogenesis, Co-cultured, Human adipose-derived stem cells, Nasal chondrocytes, Three-dimensional system, Cartilage repair

## Abstract

**Background:**

The three-dimensional (3D) system is one of the important factors to engineer a biocompatible and functional scaffold for the applications of cell-based therapies for cartilage repair. The 3D alginate hydrogels system has previously been shown to potentially promote chondrogenesis. The chondrocytic differentiation of co-cultured adipose-derived stem cells (ADSCs) and nasal chondrocytes (NCs) within alginate constructs are hypothesized to be influenced by concentration of alginate hydrogel. In this study, we evaluated the effects of alginate concentration on chondrogenic differentiation of ADSCs and NCs co-cultured in a biological approach.

**Method:**

The co-cultured cells of 2:1 ADSCs-to-NCs ratio were encapsulated in alginate constructs in one of three concentrations (1.0%, 1.2% and 1.5%) and cultured under serum free conditions for 7 days. Cell viability, cell proliferation, immunohistochemical, gycosaminogylycans (GAG) synthesis, and gene expression were examined.

**Results:**

Overall, the 1.2% alginate concentration group was relatively effective in chondrocytic differentiation in comparable to other groups. The cell morphology, cell viability, and cell proliferation revealed initial chondrogenic differentiation by the formation of cell clusters as well as the high permeability for exchange of solutes. The formation of newly synthesis cartilage-specific extracellular matrix in 1.2% group was demonstrated by positive immunohistochemical staining of collagen type II. The co-cultured cells in 1.2% group highly expressed COL II, ACP and SOX-9, compared to 1.0% and 1.5% groups, denote the retention of cartilaginous-specific phenotype by suppressing the undifferentiation stem cell markers of SOX-2 and OCT-4. The study showed 1.2% group was less likely to differentiate towards osteogenesis by downregulating hyperthrophy chondrocytic gene of COL X and osseous marker genes of OSC and OSP.

**Conclusion:**

This study suggests that variations in the alginate concentration of co-cultured ADSCs and NCs influenced the chondrogenesis. The remarkable biological performance on chondrogenic differentiation in regulating the concentration of alginate 3D culture provides new insights into the cell cross-talk and demonstrates the effectiveness in regenerative therapies of cartilage defects in tissue engineering.

## Background

Injectable alginate hydrogels system has recently raised the interest in tissue engineering and regenerative medicine. The challenges such as the invasive implantation procedure and poor cell retention within the defect can be improved using this system [1–3]. The injectable delivery system allows cells encapsulated in hydrogels to exactly fill tissue voids and complex defects in the articular surface [4]. The three-dimensional (3D) system plays a crucial role leading to a successful injectable alginate hydrogels system with the recent advancement of cell-based tissue engineering application in cartilage regeneration. Culturing cells in 3D environment is well known for cell cross-talk and cell-to-extracellular matrix (ECM) interactions for initial chondrocytic differentiation and maintain of chondrogenic phenotype [5]. 3D culture mimics the in vivo microenvironment to promote strong cellular interactions. Recent studies demonstrated superior chondrogenesis by co-culturing mesenchymal stem cells (MSCs) and articular chondrocytes in 3D culture environment than monolayer culture [6–8]. However, the co-cultured cells in 3D microenvironment with direct cellular interactions has the tendency to form heterokaryons [9].

The 3D alginate hydrogels system has been proposed to physically immobilize the co-cultured cells via minimal direct cell contact [10]. Alginate has been extensively used as a biocompatible carrier for cartilaginous regeneration in tissue engineering approaches. Encapsulation of cells in alginate is capable to crosslink with calcium to form hydrogels and function at the physiological conditions. The alginate properties of natural hydrocolloidal in gelation tend to form a stable quasi-solid structure with high porosity and water content. The pore structure is highly dependent on the concentration of hydrogel. Theoretically the increase in concentration of hydrogels may reduce in efficiency of solutes transportation and nutrient supply and therefore, leading to a lower diffusion coefficient [11]. Several studies indicated the transport of solutes is concentration dependence, which the diffusion coefficient decreased with higher concentration of alginate gel [12, 13].

To date, there are very few studies on the co-cultured system using alginate constructs as the biomaterial for cartilaginous regeneration in cell-based tissue engineering. A comparative study evaluated the chondrogenic differentiation potential of a single cell type supplemented with growth factors [14]. Some reports focused mainly on the mechanical properties of alginate constructs with or without cells [15–17]. However, there are no quantitative and systematic study especially on the concentration of alginate hydrogels as a scaffold material to drive chondrogenesis of MSCs in a co-culture model on a biological approach. Cell sources remained as one of the factors that are pivotal to the success of chondrocytic differentiation via co-culture system. Adipose-derived stem cells (ADSCs) are widely known to be an attractive alternative source of adult stem cells which is capable of self-renewal and proliferate in a large degree with its high telomerase activity [18, 19], while nasal septum cartilage or nasal chondrocytes (NCs) serve as a great alternative source of non-articular cartilage that eliminated the issue of donor morbidity and still preserve the chondrocytic phenotype [20]. ADSCs culture in alginate hydrogels have previously been proven to promote chondrogenic differentiation [21, 22]. The chondrogenic differentiation of ADSCs can be increased with appropriate ratio of chondrocytes in co-culture and the cell seeding density [23]. However, another crucial issue for ADSCs chondrogenesis in alginate hydrogels is the gel concentration because it affects the complex interaction of growth and differentiation factors as well as nutrient and waste flow. Therefore, the interest raised on the study to determine the most effective concentration of alginate hydrogel to provide the appropriate physiological conditions of microenvironment for chondrogenic differentiation by co-culturing ADSCs and NCs. In this study, the biological performance on the efficacy of alginate hydrogel concentration on chondrocytic differentiation with encapsulation of co-cultured ADSCs and NCs have been investigated.

## Methods

### Harvesting and expansion of human adipose-derived stem cells (ADSCs)

The human abdominal subcutaneous adipose tissues were harvested from patients (*n* = 6, where *n* is sample size) with written informed consent after caesarean section and all the experimental procedures were approved by the Research Ethics Committee Universiti Kebangsaan Malaysia (FF-2015-220). The adipose tissue was minced into small pieces. These pieces were then digested with 0.3% collagenase type I (Worthington Biochemical Corporation, NJ, USA) for an hour in an orbital shaker incubator at 37 °C. The supernatant was aspirated and the tissue was washed twice with phosphate-buffered saline (PBS; pH 7.22, Gibco, NY, USA). The isolated ADSCs were resuspended in complete medium (Ham’s F12 and Dulbecco’s modified Eagle medium (DMEM/F-12; Gibco) containing 10% fetal bovine serum (FBS; Gibco), 1% antibiotic-antimycotic (Gibco), 1% glutamax (Gibco), and 1% of 50 μg/ml ascorbic acid (Sigma-Aldrich, St. Louis, USA)) and incubated in vitro to confluence. The medium was changed every 48 h. The cells were trypsinized using 0.125% trypsin-ethylene diamine tetra acetic acid (EDTA; Gibco) and subcultured until passage 3 to 5 for encapsulation in alginate constructs.

### Harvesting and expansion of human nasal Chondrocytes (NCs)

The nasal septum cartilage was harvested from six patients (*n* = 6) who underwent an elective septoplasty procedure with written informed consent (approved by Research Ethics Committee Universiti Kebangsaan Malaysia with approval ethic code, FF-2015-220). The septal cartilage specimen was diced into fine pieces. The diced cartilage were digested using 0.6% collagenase type II (Worthington Biochemical Corporation, NJ, USA) in an orbital shaker incubator for 4 h at 37 °C. The supernatant was discarded and the cell pellet was washed with PBS. The isolated NCs were cultured in complete medium (as described in harvesting and expansion of ADSCs) and incubated at 5% CO_2_ at 37 °C. The medium was changed every other day. The cells were trypsinized using 0.125% trypsin-EDTA and then proceeded with encapsulation for chondrogenic differentiation.

### Alginate encapsulation of co-culture ADSCs and NCs

The powder form of sodium alginate (Sigma-Aldrich, USA) was weighed accordingly and dissolved in 0.9% *w*/*v* sodium chloride solution to form aqueous solution with the concentrations of 1.0%, 1.2%, and 1.5%. Three million cells comprising of ADSCs and NCs were co-cultured in 2:1 ratio. The cell ratio of 2:1 was the optimum co-culture ratio as previously determined to promote chondrogenesis [23]. The mixture of cells were resuspended in alginate solution to a final cell density of 3 × 10^6^ cells/ml and the final alginate concentrations respectively as previously described [24, 25]. The suspension was pressed through a syringe with 27-gauge needle in a droplet method into the 102 mM calcium chloride solution that initiated gelation and formed spherical alginate-cell constructs. The alginate-cell constructs were maintained for 8 min in 102 mM calcium chloride solution to complete the polymerization process. The constructs were then filtered using cell strainer and were rinsed with 0.9% sodium chloride solution and serum free medium (Ham’s F12 and Dulbecco’s modified Eagle medium (DMEM/F-12; Gibco) supplemented with 1% Insulin-Transferring-Selenium-X Supplement (ITS; Gibco), 1% antibiotic-antimycotic, 1% glutamax, and 1% ascorbic acid) successively. The microencapsulated cells were cultured in suspension cultures for 7 days with serum free medium. The medium was replaced every alternate day.

### Morphological assessment and cell viability

The alginate-cell constructs were observed with an imaging system (EVOS FL Colour; Life Technologies, USA) and the morphology of encapsulated cells were photographed at day 7. The viability of the co-cultured cells in the alginate hydrogel was evaluated with trypan blue exclusion test using a haemocytometer.

### Immunohistochemical analysis

After 7 days in culture, the constructs were fixed in 10% formalin and embedded into paraffin. Sections with a thickness of 5 μm were cut. The presence of collagen type II was immunolocalized with mouse monoclonal antibody against collagen type II (Thermo Scientific, USA). After 30 min of room temperature incubation with primary antibody, sections were incubated with rabbit anti-mouse secondary antibody (DAKO, Denmark) for another 30 min. This is followed by positive staining visualization using 3,3′-Diaminobenzidine (DAB; DAKO) and counterstained nucleus in hemotoxylin. Microscopic images were photographed with a microscopy imaging system (Q550IW; Leica, Germany).

### Cell proliferation

Deoxyribonucleic acid (DNA) content of microencapsulated cells was quantified at day 7 for cell proliferation assessment. The samples were purified using PureLink Genomic DNA mini kit (Life Technologies, USA). Total DNA of digested sample was quantified using UV-Vis spectrophotometer (NanoDrop 2000; Thermo Scientific, USA).

### Biochemical analysis

Glycosaminoglycan (GAG) is the major component of cartilage extracellular matrix. After 7 days of culture, the synthesis of GAG was evaluated by the 1,9-dimethymethylene blue (DMMB; Biocolor, UK) method. The samples were digested in papain lysis buffer (Sigma-Aldrich, USA) for 3 h at 65 °C. The amount of GAG accumulation in alginate-cell construct was tabulated by calibrating against a standard curve constructed from bovine tracheal chondroitin-4-sulfate (Biocolor, UK). The absorbance was detected using a microplate spectrophotometer (Thermo Scientific, USA) at a wavelength of 595 nm. The GAG content was normalized to the DNA content to examine the biosynthetic activity of microencapsulated cells.

### RNA extraction and gene expression

Real-time polymerase chain reaction (RT-PCR) was used to quantify the expression of targeted genes in each sample at day 7. The samples were digested using TRIzol reagent (Invitrogen, USA) and the total RNA were extracted. Total RNA was transcribed into single-strand cDNA using SuperScript III First-strand synthesis kit (Life technologies, USA) in accordance to manufacturer’s recommendations. Table 1 shows the genes for RT-PCR analysis: chondrogenic genes were used as positive gene expressions, stemness genes were served as negative control, and osteogenic genes were used to regulate osteogenesis. All targeted genes were normalized to the endogenous housekeeping gene, Glyceraldehyde-3-phosphate dehydrogenase (GAPDH). The PCR reaction was evaluated using the SYBR Select Master Mix (Life technologies, USA) with a RT-PCR machine (iCycler; Bio-Rad, USA). The effects of cartilaginous phenotype restoration were analyzed using COL II-to-COL I ratio.

**Table 1:** List of primer sequences for RT-PCR analysis

Gene	Primer 5′ - 3′	Accession no.
GADPH	F: 5′-tcc ctg agc tga acg gga ag-3′ R: 5′-gga gga gtg ggt gtc gtc gct gt-3′	NM_002046
Human Collagen Type I, COL I	F: 5′-agg gct cca acg aga tcg aga-3′ R: 5′-tac agg aag cag aca ggg cca-3′	NM_000088
Human Collagen Type II, COL II	F: 5′-cta tct gga cga agc agc tgg ca-3′ R: 5′-atg ggt gca atg tca atg atg g-3′	NM_001844
Human Aggrecan Core Protein, ACP	F: 5′-cac tgt tac cgc cac ttc cc-3′ R: 5′-acc agc gga agt ccc ctt cg-3′	NM_001135
Human Transcription Factor SOX-9, SOX-9	F: 5′-gcg gag gaa gtc ggt gaa ga-3′ R: 5′-ccc tct cgc ttc agg tca gc-3′	NM_000346
Homeobox Transcription Factor, NANOG	F: 5′- ctg tga ttt gtg ggc ctg aa −3′ R: 5′- tgt ttg cct ttg gga ctg gt-3′	NM_024865
SRY-related HMG-Box-2, SOX-2	F: 5′- tta cct ctt cct ccc act cca −3′ R: 5′- ggt agt gct ggg aca tgt gaa-3’	NM_003106
Reduced Expression-1, REX-1	F: 5′-aaa ggt ttt cga agc aag ctc-3′ R: 5′-ctg cga gct gtt tag gat ctg-3′	NM_174900
Octamer-Binding Protein 3/4, OCT-4	F: 5′- aag gat gtg gtc cga gtg tg-3′ R: 5′- gaa gtg agg gct ccc ata gc-3′	NM_002701
Osteocalcin, OSC	F: 5′-gtg cag agt cca gca aag gt −3′ R: 5′-tca gcc aac tcg tca cag tc −3′	NM_199173
Osteopontin, OSP	F: 5′-tga aac gag tca gct gga tg-3′ R: 5′-tga aat tca tgg ctg tgg aa-3′	NM_001040060
Human Collagen Type X, COL X	F: 5′-gct aag ggt gaa agg ggt tc-3′ R: 5′-ctc cag gat cac ctt ttg ga-3′	NM_000493

### Statistical analysis

The data was expressed as mean ± standard deviation. Statistical analyses of cell viability, total DNA, GAG content, and RT-PCR were analyzed by paired student’s t test in all study groups. The difference was considered significant when *P* < 0.05.

## Results

### Cell viability

**Fig. 1:**
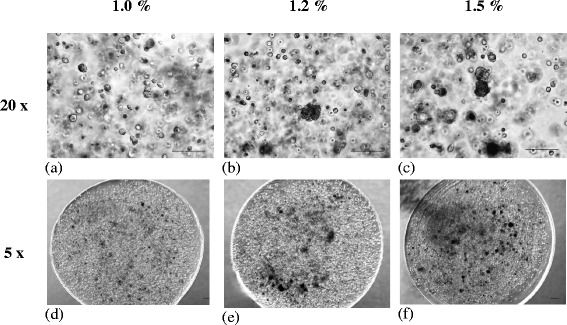
Morphological appearance of various concentrations of alginate constructs encapsulated with co-cultured ADSCs and NCs. **a** Study group with 1.0% of alginate concentration under magnification of 20×. **b** Study group with 1.2% of alginate concentration under magnification of 20×. **c** Study group with 1.5% of alginate concentration under magnification of 20×. **d** Study group with 1.0% of alginate concentration under magnification of 5×. **e** Study group with 1.2% of alginate concentration under magnification of 5×. **f** Study group with 1.5% of alginate concentration under magnification of 5×

**Fig. 2:**
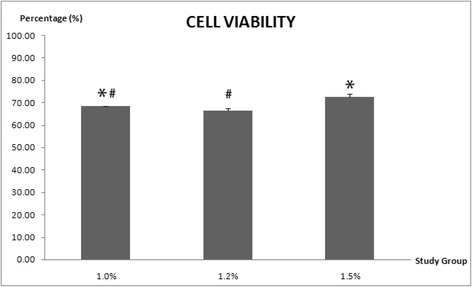
The cell viability of alginate constructs with different concentrations after 7 days of culture

The microscopic images (Fig. 1a-c) illustrated the plump and rounded morphology of co-cultured ADSCs and NCs encapsulated in alginate constructs with various concentrations. The size of alginate constructs remained spherical shape in all groups during a cultivation period of 7 days (Fig. 1d-f). The cells were sparsely and evenly distributed throughout the transparent matrix constructs. Cell clusters were found in all constructs with various concentrations, especially for the condition with the higher alginate concentration of 1.2% and 1.5%, in which the number of cell clusters was greater. The cell viability of ADSCs and NCs with co-culture ratio 2:1 in alginate construct were significant higher in 1.5% group and followed by 1.0% and 1.2% groups at day 7 (Fig. 2). All alginate concentration groups maintained high viability rate, ranging from 66% to 72% in which the differences were relatively narrow.

### Production of cartilaginous extracellular matrix

**Fig. 3:**
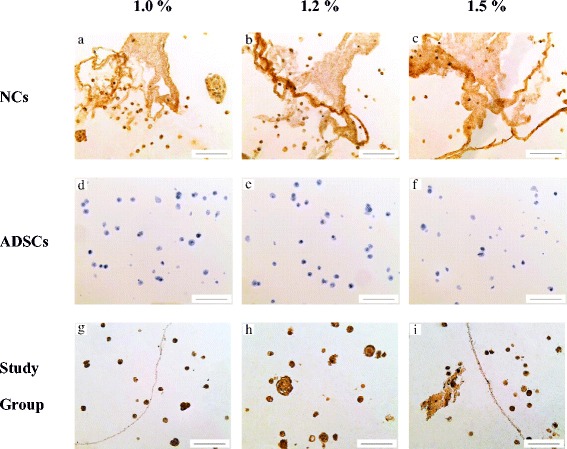
Immunohistochemical staining of ADSCs co-cultured with NCs in alginate constructs of various concentrations. **a** NCs with 1.0% of alginate concentration. **b** NCs with 1.2% of alginate concentration. **c** NCs with 1.5% of alginate concentration. **d** ADSCs with 1.0% of alginate concentration. **e** ADSCs with 1.2% of alginate concentration. **f** ADSCs with 1.5% of alginate concentration. **g** Study group with 1.0% of alginate concentration. **h** Study group with 1.2% of alginate concentration. **i** Study group with 1.5% of alginate concentration

The presence of collagen type II was detected by immunohistochemical staining. Positive staining for collagen type II was observed with all alginate concentrations groups (Fig. 3). Sections were stained on single cells and small cell clusters. Consistent with cell morphological assessment, the cells encapsulated in alginate constructs were exhibited in round shape and were slightly more intensely aggregated in 1.2% and 1.5% of alginate concentration groups. The formation of lacunae revealed the synthesis of ECM was largely observed in 1.2% group compared to other groups.

### Production of DNA and GAG contents

**Fig. 4:**
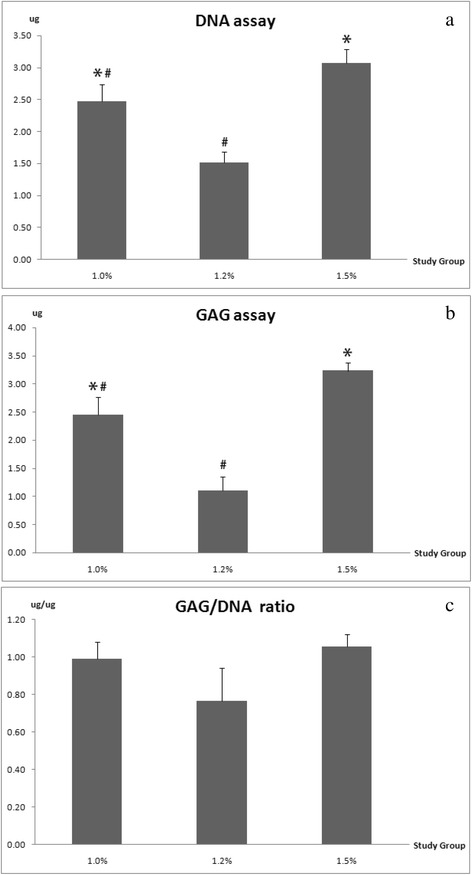
DNA and GAG content in alginate constructs with different concentrations were quantified respectively. **a** Amount of DNA content. **b** Synthesis of GAG. **c** Normalization of GAG content to DNA content

The DNA content indicated the proliferation rate of co-cultured ADSCs and NCs encapsulated in alginate constructs with increasing concentrations. The amount of DNA was significantly higher in alginate concentration of 1.5% group comparable to other groups after cultured for 7 days, whereas 1.0% group has a lower cellularity and followed by 1.2% group (Fig. 4a). This cell proliferation data was responded to the cell viability rate, in which an increase in cell viability with the highest alginate concentration group. The amount of GAG content was quantified by DMMA assay throughout cultivation period of 7 days. The newly synthesis GAG contents of 1.5% alginate concentration group has a significant increase compared to other lower alginate concentration groups (Fig. 4b). The finding of GAG synthesis was in agreed with the amount of DNA content.

The amount of GAG content was normalized to amount of DNA content (Fig. 4c) to demonstrate differences in the biosynthesis activity of the co-cultured cells among the groups with various alginate concentrations. The normalization of GAG/DNA revealed similar trends in all groups, however there was no significant differences after normalization in each group.

### Gene expression analysis

**Fig. 5:**
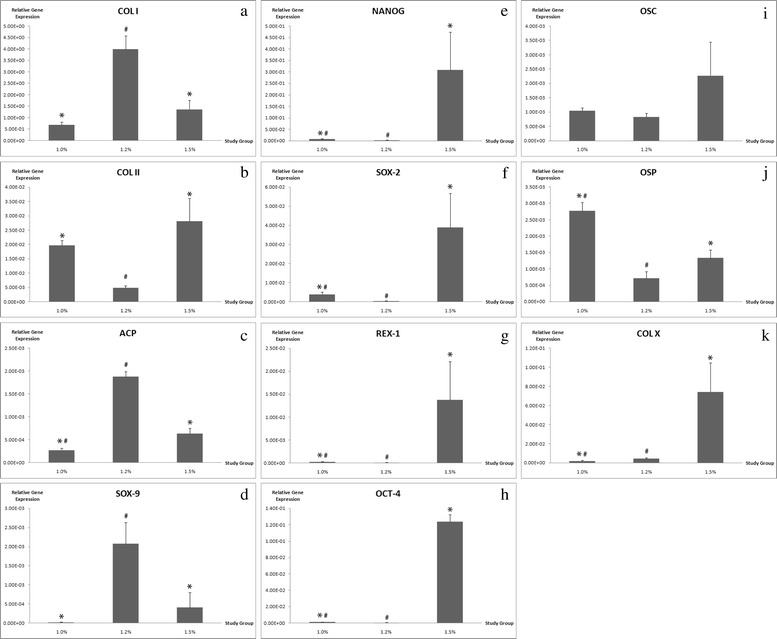
Quantitative real-time gene expressions analysis of **a**-**d** chondrogenic genes, **e**-**h** stemness genes, and **i**-**k** osteogenic genes. **a** COL I, **b** COL II, **c** ACP, **d** SOX-9, **e** NANOG, **f** SOX-2, **g** REX-1, **h** OCT-4, **i** OSC, **j** OSP, **k** COL X

The co-cultured of ADSCs and NCs were encapsulated in alginate constructs with different alginate concentrations were analyzed. The alginate concentration of 1.2% group demonstrated an overall remarkable upregulation of chondrogenic marker genes in comparison to 1.5% concentration group at day 7 (Fig. 5b-d). The 1.2% group showed a a nearly 300-fold induction of ACP expression. The cartilaginous markers of SOX-9 was highly expressed in 1.2% group and showed a significant increase by 516-fold respectively. Conversely, the expression of stemness gene markers was strongly downregulated in 1.2% alginate concentration group. The gene expression of NANOG, SOX-2, REX-1 AND OCT-4 were significantly higher in 1.5% group after cultured for 7 days, whereas 1.0% group maintained at a lower level in all stemness markers (Fig. 5e-h). The osteogenic markers of OSC, OSP and COL X were analyzed to evaluate the osteogenesis tendency of co-cultured ADSCs and NCs in alginate constructs. The co-cultured cells in 1.2% alginate concentration group showed a low level of expression in osteogenic genes (Fig. 5i-k). However, the 1.0% and 1.5% groups significantly upregulated OSP and COL X at day 7.

**Fig. 6:**
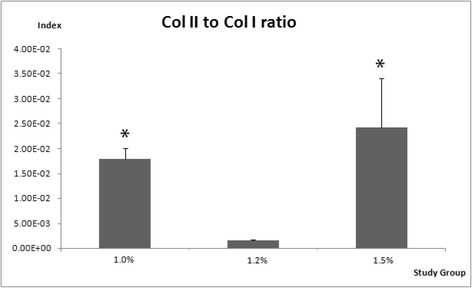
Quantitative analysis of cartilaginous phenotype restoration index of COL II-to-COL I

COL II was normalized to COL I to analyze the restoration index of cartilaginous phenotype among different alginate concentration groups as showed in Fig. 6. The COL II-to-COL I ratio demonstrated the significant differences in 1.2% group in relative to 1.0% and 1.5% groups.

## Discussion

Recent studies reported alginate is an established and biocompatible hydrogel scaffold material that is capable for cartilaginous regeneration [16, 26]. However, there is no quantitative biological study on the effects of alginate concentration in chondrogenic differentiation of co-cultured system. Also, current commercially available cellular based therapy required a long cultivation period to prepare sufficient amount of chondrocytes for transplantation [27, 28]. Thus, our study aimed to develop early chondrogenic progenitor cells achieved by 3D alginate co-cultured system to shorten the culture period when comes to clinical application. In this study, we evaluated the efficacy of chondrogenic pre-differentiation when encapsulated co-cultured ADSCs and NCs in the ratio of 2:1 in alginate constructs with different concentrations. All the study groups were evaluated at day 7 as previous study shown that cells showed evident of chondrogenesis as early as 7 days by forming dense spherical cellular bodies with the setting [29].

The ADSCs and NCs exhibited a spherical morphology when co-cultured in the three-dimensional alginate hydrogels even in serum free culture condition. The co-cultured cells encapsulated in alginate constructs underwent an instant chondrogenic switch to recover their cartilaginous-specific phenotype [30, 31]. The co-cultured cell aggregates and formed cell clusters at day 7 demonstrated the formation of early chondrogenic differentiation especially in 1.2% and 1.5% groups. Previous studies reported the formation of functional tissue-engineered cartilage tissue initiated with the formation of cellular aggregates as early as cultivation period of 5 days [29, 32]. The cell viability in alginate constructs with various concentrations was relatively high after cultivation period of 7 days. The high percentage of viable cells indicated the high permeability of the alginate constructs that allows adequate amount of nutrient supply to the cells even in the serum free cultured condition. The cross-linking of alginate-cells by calcium creates a highly hydrated three-dimensional microenvironment at physiological conditions, in which the cells encapsulated in alginate constructs are highly functional and have a high cellularity [33].

The cartilage-specific extracellular matrix (ECM) of collagen type II was deposited within the alginate constructs among different concentration of alginate groups from immunohistochemical analysis. The production of ECM is relatively high by alginate concentration of 1.2% group with the observation of lacunae synthesis. The results were consistent with the morphological assessment, in which the aggregation of cells and formation of cell clusters were observed denotes the production of cartilaginous matrix to form early chondrogenesis.

In this study, the amount of DNA denotes the quantitative analysis of cell proliferation. Since the cells were immobilized in the alginate constructs, we would not expect significant increase in cell proliferation [25, 34]. Consistent with the findings in this study, the overall amount of DNA in each study group is within the moderate range. However, we did note that the lower DNA content in 1.2% group had much higher viability of cells, which is consistent as the previous findings [26]. The GAG content exhibited a similar trend which lower amount of cartilaginous matrix was observed in 1.2% group than other groups. This is possibly due to the encapsulated co-cultured cells are in the initial stage of cellular condensation process in 1.2% alginate constructs [14]. The normalization of GAG-to-DNA showed significant differences in 1.2% group which demonstrates the biosynthesis activities were initiated at this concentration compared to other concentration groups. Although 1.0% and 1.5% groups exhibited higher cell proliferation and GAG content, the gene expressions in both study groups demonstrated a low level of chondrogenic gene expression.

The quantitative analysis of real-time PCR was used to study the gene expressions patterns in various concentration of alginate constructs. The co-cultured of ADSCs and NCs showed an expression of chondrocyte-specific marker genes such as ACP, SOX-9, collagen type II and collagen type I. With regard to alginate concentration, the 1.2% group expressed high level of the chondrogenic genes, whereas other groups remained low level of expressions. The higher expression of ACP represent the newly synthesis of cartilaginous ECM [35, 36]. The early markers for chondrogenesis such as COL I serve as the most abundant collagen in human body [37] and SOX-9 act as a transcription factor to regulate the other gene expression involved in chondrogenic differentiation [38]. In contrast, the stemness markers were highly upregulated in 1.5% group comparable to the significantly downregulated 1.2% group, which denotes the co-cultured cells in 1.5% group preserve most of the stemness properties such as NANOG and REX-1 impose the pluripotency on stem cells and remained undifferentiated [39]. SOX-2 is the transcription regulators that forms a trimeric complex with OCT-4 to regulate the number of stemness gene expressions [40], while OCT-4 is commonly used as an undifferentiated stem cells marker due to the self-renewal ability of undifferentiated stem cells [41]. To exclude the unwanted osteogenic differentiation of the cultured cells, OSC and OSP was analyzed. The expression of osseous marker genes were decreased significantly in 1.2% group compared to 1.5% group, whereas 1.0% group exhibited higher osteogenic markers as well. OSC is one of the osteoblast specific gene and OSP is responsible for bone mineralization [42, 43]. This suggests higher GAG content in 1.0% group has the tendency moving towards osteogenesis. The promotion of Collagen type X indicates hypertrophic chondrocytes that may led the co-cultured cells to eventually differentiated towards osteogenesis [44]. Downregulated of COL X in 1.2% group compared to 1.5% group indicates minimal hypertrophy as COL X is not favorable in ECM of hyaline cartilage [45]. The overall quantitative mRNA expression profile suggests the alginate concentration of 1.2% group yielded the most favourable chondrogenic differentiation when encapsulated with co-cultured ADSCs and NCs by maintaining the healthy chondrocytic phenotype without the tendency to differentiate into bone.

This study demonstrated the 1.5% group exhibited higher synthesis of ECM, however the gene expression shows that higher stemness properties are also found within this group, in which suggesting most of the ADSCs were remained undifferentiated and the high matrix production may secrete mainly by NCs than ADSCs.

The properties of alginate constructs are dependent on the process of cross-linking. The concentration of alginate solution is one of the factor which influence the stiffness or elasticity of the alginate constructs after gelation [33]. The current study suggests the lowest alginate concentration of 1.0% was too lowand the highest alginate concentration of 1.5% was quite stiff to induce proper chondrocytic differentiation when compared to 1.2% group. The alginate constructs having an optimum concentration of 1.2% resulted from the cartilage-like cell morphology, high cell viability, synthesis of new ECM, and upregulation of chondrogenic marker genes, give rise to a stiffer and more stable hydrogel scaffold biomaterial. It has been studied in the recent literature that the gold standard of cartilage regeneration therapeutic approaches for encapsulation of cell culture in alginate hydrogel is 1.2%, in which support the findings of the current study [46, 47].

## Conclusion

In summary, these findings are quite evident that the variations in concentrations of alginate constructs by co-culturing ADSCs and NCs influences the cartilaginous differentiation. Our data suggests alginate constructs in different concentrations create a microenvironment that influenced the chondrocytic differentiation with support of cell viability and gene expressions. This study contributes to better understanding of the effects of alginate concentration in the aspect of stem cell differentiation, suggesting the potential application in cartilage tissue engineering.

## Abbreviations

3D: Three-Dimensional
ACP: *Human Aggrecan Core Protein*
ADSCs: Adipose-Derived Stem Cells
COL I: *Human Collagen Type I*
COL II: *Human Collagen Type II*
COL X: *Human Collagen Type X*
DAB: 3,3′-Diaminobenzidine
DMEM/F-12: Ham’s F12 and Dulbecco’s Modified Eagle Medium
DMMB: 1,9-Dimethymethylene Blue
DNA: Deoxyribonucleic Acid
ECM: Extracellular Matrix
EDTA: Trypsin-Ethylene Diamine Tetra Acetic Acid
FBS: Fetal Bovine Serum
GAG: Gycosaminogylycans
GAPDH: Glyceraldehyde-3-Phosphate Dehydrogenase
ITS: Insulin-Transferring-Selenium-X
MSCs: Mesenchymal Stem Cells
NANOG: *Homeobox Transcription Factor*
NCs: Nasal Chondrocytes
OCT-4: *Octamer-Binding Protein 3/4*
OSC: *Osteocalcin*
OSP: *Osteopontin*
PBS: Phosphate-Buffered Saline
REX-1: *Reduced Expression-1*
RT-PCR: Real-Time Polymerase Chain Reaction
SOX-2: *SRY-related HMG-Box-2*
SOX-9: *Human Transcription Factor SOX-9*

## References

[1] Sun J, Tan H (2013). Alginate-based biomaterials for regenerative medicine applications. Materials.

[2] Bidarra SJ, Barrias CC, Granja PL (2014). Injectable alginate hydrogels for cell delivery in tissue engineering. Acta Biomater.

[3] Lum L, Elisseeff J. Chapter 4: Injectable hydrogels for cartilage tissue engineering. http://www.oulu.fi/spareparts/ebook_topics_in_t_e/abstracts/lum_01.pdf (2003). Accessed 3 Mar 2017.

[4] Stevens M (2004). A rapid-curing alginate gel system: utility in periosteum-derived cartilage tissue engineering. Biomaterials.

[5] Yu D, Han J, Kim B (2012). Stimulation of chondrogenic differentiation of mesenchymal stem cells. Int J Stem Cells.

[6] Meretoja VV, Dahlin RL, Kasper FK, Mikos AG (2012). Enhanced chondrogenesis in co-cultures with articular chondrocytes and mesenchymal stem cells. Biomaterials.

[7] Diekman BO, Rowland CR, Lennon DP, Caplan AI, Guilak F (2010). Chondrogenesis of adult stem cells from adipose tissue and bone marrow: induction by growth factors and cartilage-derived matrix. Tissue Eng Part A.

[8] Cooke M, Allon A, Cheng T, Kuo A, Kim H, Vail T (2011). Structured three-dimensional co-culture of mesenchymal stem cells with chondrocytes promotes chondrogenic differentiation without hypertrophy. Osteoarthritis Cartilage.

[9] Ying QL, Nichols J, Evans EP, Smith AG (2002). Changing potency by spontaneous fusion. Nature.

[10] Mo XT, Guo SC, Xie HQ, Deng L, Zhi W, Xiang Z (2009). Variations in the ratios of co-cultured mesenchymal stem cells and chondrocytes regulate the expression of cartilaginous and osseous phenotype in alginate constructs. Bone.

[11] Scott CD, Woodward CA, Thompson JE (1989). Solute diffusion in biocatalyst gel beads containing biocatalysis and other additives. Enzyme Microb Technol.

[12] Nguyen AL, Luong JH (1986). Diffusion in kappa-carrageenan gel beads. Biotechnol Bioeng.

[13] Martinsen A, Storro I, Skjark-Braek G (1992). Alginate as immobilization material: III. Diffusional properties. Biotechnol Bioeng.

[14] Hui TY, Cheung KM, Cheung WL, Chan D, Chan BP (2008). In vitro chondrogenic differentiation of human mesenchymal stem cells in collagen microspheres: influence of cell seeding density and collagen concentration. Biomaterials.

[15] Kuo CK, Ma PX (2008). Maintaining dimensions and mechanical properties of ionically crosslinked alginate hydrogel scaffolds in vitro. J Biomed Mater Res A.

[16] Awad HA, Wickham MQ, Leddy HA, Gimble JM, Guilak F (2004). Chondrogenic differentiation of adipose-derived adult stem cells in agarose, alginate, and gelatin scaffolds. Biomaterials.

[17] Wang CC, Yang KC, Lin KH, Liu HC, Lin FH (2011). A highly organized three-dimensional alginate scaffold for cartilage tissue engineering prepared by microfluidic technology. Biomaterials.

[18] Hamid AA, Idrus RBH, Saim AB, Sathappan S, Chua KH (2012). Characterization of human adipose-derived stem cells and expression of chondrogenic genes during induction of cartilage differentiation. Clinics.

[19] V Vats A, Tolley NS, Bishop AE, Polak JM (2005). Embryonic stem cells and tissue engineering: delivering stem cells to the clinic. J R Soc Med.

[20] Homicz MR, McGowan KB, Lottman LM, Beh G, Sah RL, Watson D (2003). A compositional analysis of human nasal septal cartilage. Arch Facial Plast Surg.

[21] Galateanu B, Dimonie D, Vasile E, Nae S, Cimpean A, Costache M (2012). Layer-shaped alginate hydrogels enhance the biological performance of human adipose-derived stem cells. BMC Biotechnol.

[22] Wang T, Lai JH, Han LH, Tong X, Yang F (2014). Chondrogenic differentiation of adipose-derived stromal cells in combinatorial hydrogels containing cartilage matrix proteins with decoupled mechanical stiffness. Tissue Eng Part A.

[23] Ewa-Choy YW, Murphy BP, Nur-Azurah AG, Jahendran J, Chua KH (2016). Effect of cell ratio on chondrogenesis of co-cultured human adipose-derived stem cells and nasal chondrocytes in alginate hydrogel. Regener Res.

[24] Wang H, Zhou Y, Huang B, Liu LT, Liu MH, Wang J (2014). Utilization of stem cells in alginate for nucleus pulposus tissue engineering. Tissue Eng Part A.

[25] Schmitt A, Rodel P, Anamur C, Seeliger C, Imhoff AB, Herbst E, et al. Calcium alginate gels as stem cell matrix-making paracrine stem cell activity available for enhanced healing after surgery. Plos One. 2015; 10.1371/journal.pone.0118937. 10.1371/journal.pone.0118937 PMC4368733 25793885

[26] Endres M, Wenda N, Woehlecke H, Neumann K, Ringe J, Erggelet C (2010). Microencapsulation and chondrogenic differentiation of human mesenchymal progenitor cells from subchondral bone marrow in Ca-alginate for cell injection. Acta Biomater.

[27] Vinatier C, Bouffi C, Merceron C, Gordeladze J, Brondello JM, Jorgensen C (2009). Cartilage tissue engineering: towards a biomaterial-assisted mesenchymal stem cell therapy. Curr Stem Cell Res Ther.

[28] Neri S, Mariani E, Cattini L, Facchini A (2011). Long-term in vitro expansion of osteoarthritic human articular chondrocytes do not alter genetic stability: a microsatellite instability analysis. J Cell Physiol.

[29] Bhumiratana S, Eton RE, Oungoulian SR, Wan LQ, Ateshian GA, Vunjak-Novakovic G (2014). Large, stratified, and mechanically functional human cartilage grown in vitro by mesenchymal condensation. Proc Natl Acad Sci U S A.

[30] Schulze-Tanzil G, de Souza P, Villegas Castrejon H, John T, Merker HJ, Scheid A (2002). Redifferentiation of dedifferentiated human chondrocytes in high-density cultures. Cell Tissue Res.

[31] Yates KE, Allemann F, Glowacki J (2005). Phenotypic analysis of bovine chondrocytes cultured in 3D collagen sponges: effect of serum substitutes. Cell Tissue Bank.

[32] Oka Y, Sato Y, Tsuda H, Hanaoka K, Hirai Y, Takahashi Y (2006). Epimorphin acts extracellularly to promote cell sorting and aggregation during the condensation of vertebral cartilage. Dev Biol.

[33] Andersen T, Strand BL, Formo K, Alsberg E, Christensen BE. Alginates as biomaterials in tissue engineering. In: Carbohydrate Chemistry. Vol. 37. 2011. p. 227–258. doi: 10.1039/9781849732765-00227.

[34] Andersen T, Auk-Emblem P, Dornish M (2015). 3D Cell Culture in Alginate Hydrogels. Microarrays.

[35] Chen CW, Tsai YH, Deng WP, Shih SN, Fang CL, Burch JG (2005). Type I and II collagen regulation of chondrogenic differentiation by mesenchymal progenitor cells. J Orthop Res.

[36] Ishiguro N, Kojima T (2004). Role of aggrecanase and MMP in cartilage degradation. Clin Calcium.

[37] Gelse K (2003). Collagens—structure, function, and biosynthesis. Adv Drug Deliver Rev.

[38] Lefrebvre V, de Crombrugghe B (1998). Toward understanding S0X9 function in chondrocyte differentiation. Matrix Biol.

[39] Fariha MM, Chua KH, Tan GC, Tan AE, Hayati AR (2011). Human chorion-derived stem cells: changes in stem cell properties during serial passage. Cytotherapy.

[40] Boyer LA, Lee TI, Cole MF, Johnstone SE, Levine SS, Zucker JP (2005). Core transcriptional regulatory circuitry in human embryonic stem cells. Cell.

[41] Niwa H, Miyazaki J, Smith AG (2000). Quantitative expression of Oct-3/4 defines differentiation, dedifferentiation or self-renewal of ES cells. Nat Genet.

[42] Nakamura A, Dohi Y, Akahane M, Ohgushi H, Nakajima H, Funaoka H (2009). Osteocalcin secretion as an early marker of in vitro osteogenic differentiation of rat mesenchymal stem cells. Tissue Eng Part C-Me.

[43] Sodek J, Ganss B, McKee MD (2000). Osteopontin. Crit Rev Oral Biol M.

[44] Shen G (2005). The role of type X collagen in facilitating and regulating endochondral ossification of articular cartilage. Orthod Craniofac Res.

[45] Herlofsen SR, Kuchler AM, Melvik JE, Brinchmann JE (2011). Chondrogenic differentiation of human bone marrow-derived mesenchymal stem cells in self-gelling alginate discs reveals novel chondrogenic signature gene clusters. Tissue Eng Part A.

[46] Guggisberg S, Benneker L, Keel M, Gantenbein B. Mechanical loading promoted discogenic differentiation of human mesenchymal stem cells incorporated in 3D–PEG scaffolds with rhGDF5 and RGD- results from International Journal of Stem cell Research and Therapy. Clin Med International Library. 2015;2(006).

[47] Swioklo S, Ding P, Pacek AW, Connon CJ. Process parameters for the high-scale production of alginate-encapsulated stem cells for storage and distribution throughout the cell therapy supply chain. Process Biochem. 2016. https://doi.org/10.1016/j.procbio.2016.06.005.

